# The preparation of an infectious full-length cDNA clone of Saffold virus

**DOI:** 10.1186/1743-422X-8-110

**Published:** 2011-03-09

**Authors:** Toshiki Himeda, Takushi Hosomi, Naeem Asif, Hiroyuki Shimizu, Takako Okuwa, Yasushi Muraki, Yoshiro Ohara

**Affiliations:** 1 Department of Microbiology, Kanazawa Medical University School of Medicine, Ishikawa, Japan; 2 The Public Health Institute of Kochi Prefecture, Kochi, Japan; 3 Department of Virology II, National Institute of Infectious Diseases, Tokyo, Japan

## Abstract

The pathogenicity of Saffold virus (SAFV) among humans still remains unclear, although it was identified as a novel human cardiovirus in 2007. In order to encourage the molecular pathogenetic studies of SAFV, we generated an infectious cDNA clone of SAFV type 3 (SAFV-3). The present study demonstrated that the synthesis of the full-length infectious RNA by T7 RNA polymerase was terminated by a homologous sequence motif with the human preproparathyroid hormone (PTH) signal in the SAFV-3 genome. To obtain the infectious RNA using T7 promoter, a variant of T7 RNA polymerase, which fails to recognize the PTH signal, was useful. This study will provide a valuable technical insight into the reverse genetics of SAFV.

## Background

The genus *Cardiovirus *belongs to the *Picornaviridae *family and is divided into two species: *Theilovirus *and *Encephalomyocarditis virus *(EMCV). *Cardiovirus *is thought to be associated with myocarditis, encephalitis and demyelinating disease in rodents [1,2]. EMCV is widely used as an experimental model for human diseases such as myocarditis, encephalitis and pancreatitis in rodents. TO subgroup strains of Theiler's murine encephalomyelitis virus (TMEV), a prototype of *Theilovirus*, serve as a mouse model for the human demyelinating disease, multiple sclerosis (MS) [3-5].

The existence of human cardiovirus has long been debated. In 2007, a novel cardiovirus, named Saffold virus (SAFV), was isolated as a human TMEV-like cardiovirus from an archived 1981 stool culture from an infant with a fever of unknown origin [6]. Subsequently, several groups identified Saffold-like cardioviruses, and eight genotypes of SAFV have been reported [6-10]. However, the pathogenicity of SAFV among humans remains unclear. In order to encourage the molecular pathogenetic studies of SAFV using a reverse genetics, the establishment of an infectious cDNA clone of SAFV is very important. In this study, we generated an infectious cDNA clone of SAFV-3 (the JPN08-404 strain), which is isolated from cerebrospinal fluid (CSF) of a patient with aseptic meningitis.

## Results and discussion

**Figure 1:**
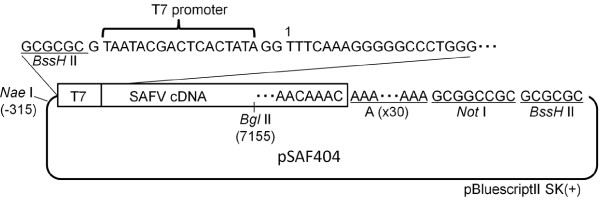
**Diagram of pSAF404 containing a full-length JPN08-404 virus cDNA**. A full-length cDNA of JPN08-404 virus (including 30 adenylate residues and *Not *I site at the 3' end) was inserted into pBluescriptII SK(+) at *Bss *HII site. The 5' end of JPN08-404 virus cDNA was placed two nucleotides downstream from the T7 promoter.

The JPN08-404 strain was isolated in LLC-MK2 from the CSF of a patient with aseptic meningitis in 2008. Enterovirus and Parechovirus were negative by PCR analysis and neutralization test in this clinical sample (data not shown). The genomes of JPN08-404 (HQ902242) and SAFV-3 (FM240787) share 97% nucleotide and 99% amino acid identity. The homology clearly indicates that JPN08-404 belongs to genotype 3 of SAFV. In this study, we generated the full-length cDNA clone of JPN08-404 by using the specific primers carrying a T7 promoter as described in **Materials and methods**. This full-length cDNA clone was designated pSAF404 (Figure 1). The RNA synthesized from pSAF404 includes some additional sequences, which are GG residues at the 5' end and GCGGCC residues past the poly (A) tract at the 3' end. These extra nucleotides of pSAF404 at the 5' and 3' ends were similar to those of infectious cDNAs of poliovirus [11] and DA strain of TMEV [12].

**Figure 2:**
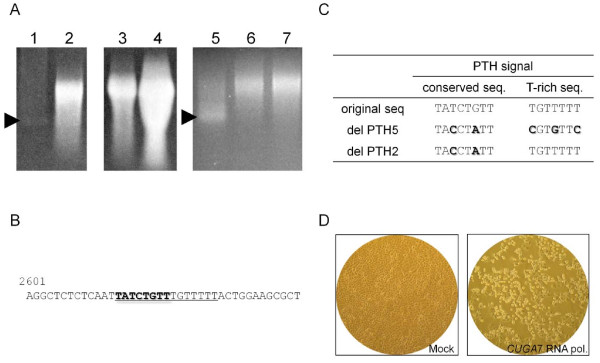
**The termination of the transcription from pSAF404 caused by the PTH signal.** A. RNAs synthesized from cDNA clones. Transcripts synthesized by Thermo T7 RNA polymerase from pSAF404 (lanes 1 and 5, arrow head) were shorter than the full-length RNA transcripts synthesized from pDAFL3, which is the infectious cDNA clone of DA strain of TMEV (lane 2). However, full-length RNAs were synthesized from pSAF404 (lane 3) and pDAFL3 (lane 4) by *CUGA *7 RNA polymerase. Full-length RNAs were also synthesized by Thermo T7 RNA polymerase from the deletion mutants of the PTH signal; delPTH2 (lane 6) and delPTH5 (lane 7). B. PTH signal in the genome of JPN08-404. An underline indicates the conserved (bold) and T-rich sequences related to the PTH signal. 2601 is the number of nucleotides. C. The mutations to delete the PTH signal. Bold letters indicate mutations. These mutations do not change the amino acid sequences. D. Cytopathic effect induced by the infection of cDNA-derived virus. Right panel: cDNA-derived JPN08-404 virus infection; left panel: mock infection.

HeLa cells were transfected with RNA, which is synthesized from pSAF404 (digested with *Not *I) by Thermo T7 RNA polymerase (TOYOBO), and the lysate of those HeLa cells was then inoculated on fresh HeLa cells. However, no cytopathic effect (CPE) was observed even after inoculating the cell lysate of these cells on other fresh HeLa cells, suggesting that the infectious viral particles were not produced from HeLa cells transfected with RNA synthesized from pSAF404. To confirm the synthesis of a full-length RNA, electrophoresis was carried out using the transcripts from pSAF404 and pDAFL3, an infectious cDNA clone of DA strain of TMEV [12] as a control (Figure 2A). The transcripts from pSAF404 were apparently shorter than those from pDAFL3 (Figure 2A, lanes 1 and 5). Therefore, the failure in the production of the infectious viral particles may be due to a premature termination during *in vitro *transcription. In order to further investigate the reason of transcriptional termination in pSAF404, the sequence of pSAF404 was compared in detail with that of pDAFL3. We identified a homologous sequence motif with the human preproparathyroid hormone (PTH) signal [13-15] within the sequence of pSAF404 (Figure 2B). The PTH signal, consisting of a conserved sequence (A/C/TATCTGTT) followed by a T rich sequence, is known as a class II site associated with the termination of transcription by bacteriophage T7 RNA polymerase [13-15]. Therefore, we next carried out the transcription from pSAF404 by using *CUGA *7 RNA polymerase (NIPPON GENE), a variant of T7 RNA polymerase which fails to recognize the PTH signal (the manufacturer's instructions, personal communication). As a result, the synthesis of full-length RNA (≒8 kb) from pSAF404 (Figure 2A, lane 3) was observed instead of the short-length RNA (Figure 2A, lane 1). However, the amount of the transcripts from pSAF404 was lower than that from pDAFL3 (Figure 2A, lanes 3 and 4). The T repeat of the 5' end of JPN08-404 may affect the transcriptional efficiency of bacteriophage RNA polymerase (the manufacturer's instructions). To further confirm whether the termination of transcription from pSAF404 is caused by the PTH signal, we generated the deletion mutants of PTH signal, delPTH2 and delPTH5 (Figure 2C). As expected, the synthesis of full-length RNAs from both delPTH2 and delPTH5 by Thermo T7 RNA polymerase were observed (Figure 2A, lanes 6 and 7). Therefore, it was demonstrated that the transcription from pSAF404 by T7 RNA polymerase was terminated by the PTH signal in the genome of JPN08-404. The amount of transcripts from delPTH5 was higher than that from delPTH2. The mutations of delPTH2 may be insufficient for the complete collapse of the PTH signal. Furthermore, the CPE was observed on HeLa cells transfected with RNA (10 μg) synthesized from pSAF404 by *CUGA *7 RNA polymerase within 48 h. Since it was not clear whether it was caused by the infectious virus particles or by the transfected RNA, the freeze-thawing lysate of these cells was inoculated to another fresh HeLa cells. The CPE was then induced on those HeLa cells (Figure 2D), indicating that the infectious virus particles were present in the lysate. In addition, the direct sequencing of the recovered virus demonstrated that the sequence of VP1 coding region is identical to that of SAFV (JPN08-404). Therefore, evasion of the termination of RNA transcription at the PTH signal is essential for the synthesis of infectious JPN08-404 RNA by T7 RNA polymerase. Among the representative SAFV strains, SAFV-3 (FM240787, GU943514) and SAFV-6 (FJ463617) possess the complete PTH signal (conserved sequence and T rich sequence) at 2614 nt, at 2415 nt and at 6516 nt, respectively, although SAFV-1 (EF165067), SAFV-2 (FN999911, EU376394, EU681176, GU943518), SAFV-3 (EU681178, HM181997) and SAFV-5 (FJ463615) do not possess the complete PTH signal. Therefore, the termination of transcription by T7 RNA polymerase may be specifically observed in some SAFV-3 and SAFV-6 strains.

**Figure 3:**
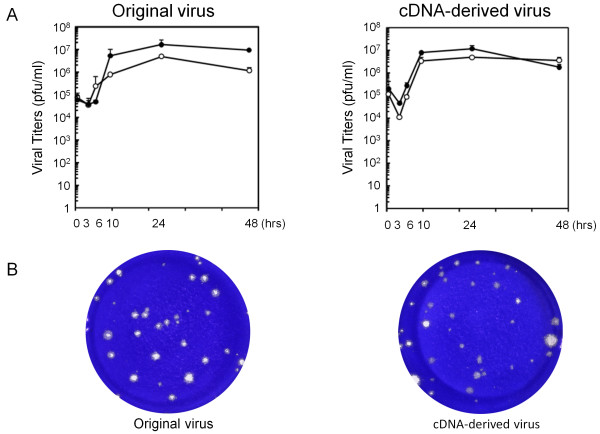
**Characteristics of the cDNA-derived JPN08-404 virus.** A: Growth kinetics of the original and cDNA-derived JPN08-404 viruses. Left panel: the original JPN08-404 virus. Right panel: the cDNA-derived JPN08-404 virus. The culture supernatants (closed circle) and the cells (open circle) were harvested at several time points indicated and assayed for titers by a standard plaque assay on HeLa cells. Titers shown are the means ± S.D. in three independent experiments. B: Plaque sizes of original and cDNA-derived JPN08-404 viruses. Left panel: the original JPN08-404 virus. Right panel: cDNA-derived JPN08-404 virus.

In the next step, growth kinetics of cDNA-derived JPN08-404 virus was analyzed by a standard plaque assay using HeLa cells. The titers of cell-free and cell-associated original JPN08-404 viruses reached a peak (1.7 × 10^7 ^and 4.8 × 10^6 ^PFU/ml, respectively) at 24 h after infection and gradually decreased thereafter (Figure 3A, left panel). The cDNA-derived JPN08-404 virus showed similar growth kinetics; reached a peak (cell-free: 1.2 × 10^7 ^PFU/ml, cell-associated: 5.0 × 10^6 ^PFU/ml) at 24 h after infection and gradually decreased thereafter (Figure 3A, right panel). The size of plaques of these viruses was almost similar (Figure 3B). These results suggested that the cDNA-derived virus has the biological activities similar to the original JPN08-404 virus.

## Conclusions

In conclusion, the infectious cDNA clone of SAFV, e.g., pSAF404 generated in present study, is thought to be a powerful tool for the molecular pathogenetic studies of SAFV using a reverse genetics. However, to obtain the infectious RNA of some SAFV-3 strains using T7 promoter, T7 RNA polymerase ignoring the PTH signal (e.g. *CUGA *7 RNA polymerase) or the deletion mutant of the PTH signal will be required. In order to generate the cDNA-derived SAFV without any artificial mutations, the combination of pSAF404 and T7 RNA polymerase, which fails to recognize the PTH signal, may be a useful tool. This study will provide a valuable technical insight into the reverse genetics of SAFV.

## Materials and methods

### Cells and viruses

HeLa cells, derived from human cervical carcinoma, were maintained in Dulbecco's modified Eagle's medium (DMEM, SIGMA) supplemented with 0.03% L-glutamine and 10% fetal bovine serum (FBS) containing 50 U/ml of penicillin and 50 μg/ml of streptomycin. LLC-MK2 cells, derived from rhesus monkey kidney epithelium, were maintained in Eagle's minimum essential medium (NISSUI) supplemented with 0.03% L-glutamine and 10% FBS containing 60 μg/ml of kanamycin. An SAFV strain, JPN08-404, was isolated in LLC-MK2 cells from the CSF (following removal of the cells by centrifugation) of a nine-year-old boy with aseptic meningitis in Kochi city, Japan, in 2008, and the isolate was identified as SAFV type 3 by sequence analysis of the VP1 region (data not shown). Thereafter, virus was propagated in HeLa cells, and the lysate was prepared by three freezing/thawing cycles to release virions. The titer of virus was determined by a standard plaque assay on HeLa cells.

### Constructions of infectious cDNA clones

To obtain a full-length clone, viral RNA was extracted from JPN08-404 propagated in HeLa cells using RNeasy mini kit (QIAGEN) according to the manufacturer's instructions. Viral RNA was reverse-transcribed by ReverTra Ace (TOYOBO) with oligo dT(20) primer. The full-length clones were generated by PCR using KOD plus Neo (TOYOBO) with the following primer set; A forward primer, 5'-CATGCGCGCGTAATACGACTCACTATAGGTTTCAAAGGGGGCCCTGGG-3', A reverse primer, 5'-CATGCGCGCGCGGCCGCGTTCTCATTTCCAATTAAAAGC-3'. The forward primer contains the sequences of *Bss *HII site, T7 promoter, spacer (GG) and 5' end of SAFV-3 (FM207487), sequentially from 5' end. The reverse primer contains the sequences of 3' end of SAFV-3 (FM207487), *Not *I site and *Bss *HII site, sequentially from 3' end. PCR products were digested by *Bss *HII and then inserted into pBluescriptII SK(+) (STRATAGENE) digested by *Bss *HII. To remove the artificial mutations inserted during PCR reaction and cloning steps, the clone named pSAFL1 was reconstructed with other two clones according to the sequence of full-length viral genome of JPN08-404 (HQ902242) determined by direct sequencing and rapid amplification of cDNA ends (RACE). However, pSAFL1 did not contain the four nucleotides (5'-AAAC-3') located in 3'end of JPN08-404 and poly (A) sequences. Therefore, in order to obtain pSAF404, the clones including the fragment from *Bgl *II site (nt 7155) to poly (A) sequence with *Not *I site were generated by 3' RACE. *Bgl *II - *Not *I fragment of pSAFL1 was then replaced with the novel *Bgl *II - *Not *I fragment including the complete 3' end and 30 adenylate residues. The deletion mutants of PTH signal were generated by PCR using the specific primers including the mutations.

### *In vitro *transcription and virus generation

pSAF404 and other plasmids (deletion mutants of the PTH signal) were linearized with *Not *I, and RNA transcripts were synthesized with Thermo T7 RNA polymerase (TOYOBO) or *CUGA *7 RNA polymerase (NIPPON GENE). pDAFL3 [12] was linearized with *Xba *I, and RNA transcripts were synthesized with Thermo T7 RNA polymerase or *CUGA *7 RNA polymerase. Then, HeLa cells were transfected with the transcripts derived from pSAF404 using Lipofectin (INVITROGEN) according to the manufacturer's instructions. The cultured cells and supernatants were collected after 48 hours, and viruses were prepared by three freezing/thawing cycles to release virions. The titers of viruses were determined by a standard plaque assay on HeLa cells.

### Kinetics of virus growth in cells

The kinetics of virus growth of the original and the cDNA-derived viruses in HeLa cells was analyzed. The cells were seeded at a density of 5 × 10^5 ^cells in a 35-mm dish. After 24 h, the cells were infected with each virus at a multiplicity of infection of 5 PFU per cell. After virus adsorption at 37°C for 60 min, the cells were washed twice with Dulbecco's phosphate buffered saline, and incubated at 37°C in DMEM with 1% FBS. The cells and supernatants were collected at 0, 3, 6, 10, 24, and 48 h after infection and the cell-associated viruses were prepared by three freezing/thawing cycles from the cells. Cell-free and cell-associated viruses were titrated by a standard plaque assay on HeLa cells.

## List of abbreviations used

CPE: cytopathic effect; CSF: cerebrospinal fluid; DMEM: Dulbecco's modified Eagle's medium; EMCV: Encephalomyocarditis virus; FBS: fetal bovine serum; PTH: preproparathyroid hormone; RACE: rapid amplification of cDNA ends; SAFV: Saffold virus; TMEV: Theiler's murine encephalomyelitis virus.

## Competing interests

The authors declare that they have no competing interests.

## Authors' contributions

THi designed and performed the experiments and drafted the manuscript. THo performed the virus isolation. NA and HS determined the viral genome sequence. YM and TO supported the experiments of growth kinetics. All authors read and approved the final manuscript. YO supervised the work and edited the final version of this manuscript.
